# Ethnic differences in cross‐sectional associations between impaired glucose regulation, identified by oral glucose tolerance test or HbA_1c_ values, and cardiovascular disease in a cohort of European and South Asian origin

**DOI:** 10.1111/dme.12895

**Published:** 2015-10-19

**Authors:** S. V. Eastwood, T. Tillin, J. Mayet, D. K. Shibata, A. Wright, J. Heasman, N. Beauchamp, N. G. Forouhi, A. D. Hughes, N. Chaturvedi

**Affiliations:** ^1^ UCL Institute of Cardiovascular ScienceUniversity College LondonLondon; ^2^ National Heart and Lung InstituteImperial College LondonLondon; ^3^ Department of RadiologyUniversity of Washington Medical CentreSeattleWAUSA; ^4^ Department of RadiologyImperial College NHS Healthcare TrustLondonUK; ^5^ MRC Epidemiology UnitUniversity of CambridgeCambridgeUK

## Abstract

**Aims:**

We contrasted impaired glucose regulation (prediabetes) prevalence, defined according to oral glucose tolerance test or HbA_1c_ values, and studied cross‐sectional associations between prediabetes and subclinical/clinical cardiovascular disease (CVD) in a cohort of European and South Asian origin.

**Methods:**

For 682 European and 520 South Asian men and women, aged 58–85 years, glycaemic status was determined by oral glucose tolerance test or HbA_1c_ thresholds. Questionnaires, record review, coronary artery calcification scores and cerebral magnetic resonance imaging established clinical plus subclinical coronary heart and cerebrovascular disease.

**Results:**

Prediabetes was more prevalent in South Asian participants when defined by HbA_1c_ rather than by oral glucose tolerance test criteria. Accounting for age, sex, smoking, systolic blood pressure, triglycerides and waist–hip ratio, prediabetes was associated with coronary heart disease and cerebrovascular disease in European participants, most obviously when defined by HbA_1c_ rather than by oral glucose tolerance test [odds ratios for HbA_1c_‐defined prediabetes 1.60 (95% CI 1.07, 2.39) for coronary heart disease and 1.57 (95% CI 1.00, 2.51) for cerebrovascular disease]. By contrast, non‐significant associations were present between oral glucose tolerance test‐defined prediabetes only and coronary heart disease [odds ratio 1.41 (95% CI 0.84, 2.36)] and HbA_1c_‐defined prediabetes only and cerebrovascular disease [odds ratio 1.39 (95% CI 0.69, 2.78)] in South Asian participants. Prediabetes defined by HbA_1c_ or oral glucose tolerance test criteria was associated with cardiovascular disease (defined as coronary heart and/or cerebrovascular disease) in Europeans [odds ratio 1.95 (95% CI 1.31, 2.91) for HbA_1c_ prediabetes criteria] but not in South Asian participants [odds ratio 1.00 (95% CI 0.62, 2.66); ethnicity interaction *P* = 0.04].

**Conclusions:**

Prediabetes appeared to be less associated with cardiovascular disease in the South Asian than in the European group. These findings have implications for screening, and early cardiovascular prevention strategies in South Asian populations.

## What's new?

- For participants of European origin, HbA_1c_ values defined as impaired glucose regulation (prediabetes) were cross‐sectionally associated with coronary heart disease, cerebrovascular disease and the composite outcome of cardiovascular disease.
- By contrast, in South Asian participants prediabetes defined according to HbA_1c_ concentration was non‐significantly associated with cerebrovascular disease only, and associations between HbA_1c_ concentration‐defined prediabetes and overall cardiovascular disease were absent and, thus significantly weaker, than those seen in the European participants.
- This suggests that current prediabetes HbA_1c_ thresholds may be inappropriate in South Asian groups as prediabetes defined in this way did not appear to confer excess cardiovascular risk in this ethnic group.

**What's new?**

- For participants of European origin, HbA_1c_ values defined as impaired glucose regulation (prediabetes) were cross‐sectionally associated with coronary heart disease, cerebrovascular disease and the composite outcome of cardiovascular disease.
- By contrast, in South Asian participants prediabetes defined according to HbA_1c_ concentration was non‐significantly associated with cerebrovascular disease only, and associations between HbA_1c_ concentration‐defined prediabetes and overall cardiovascular disease were absent and, thus significantly weaker, than those seen in the European participants.
- This suggests that current prediabetes HbA_1c_ thresholds may be inappropriate in South Asian groups as prediabetes defined in this way did not appear to confer excess cardiovascular risk in this ethnic group.

## Introduction

In parallel to the global diabetes epidemic, population surveys indicate a burgeoning prevalence of impaired glucose regulation, often termed prediabetes hyperglycaemia (12–29%, depending on definition) 1. Although it is clear that the term ‘prediabetes’ is misleading, as not all those with prediabetes will develop diabetes, it is acknowledged that this may represent a high cardiovascular disease risk state, with implications for intervention 2. It is in that latter sense that we use this term. Conventionally, prediabetic states have been defined by either post‐glucose challenge [impaired glucose tolerance (IGT)] or fasting glycaemia [impaired fasting glycaemia (IFG)] 3, but recently, guidelines for HbA_1c_‐based definitions of prediabetes have been published 2, 4.

Relatively more prediabetes is identified by HbA_1c_ than by IFG/IGT criteria in people of South Asian origin, whereas prevalence is similar by either criterion in populations of European origin 5. It is unclear how the greater prevalence of HbA_1c_‐identified prediabetes in this former group translates to cardiovascular risk, with some authors suggesting the HbA_1c_ definition of prediabetes may be less discriminative 6. As far as we are aware, this has never before been studied in South Asian populations; the majority of studies that have compared associations between prediabetes and cardiovascular disease by diagnostic criteria are in populations of European origin 7, 8, 9, 10. Moreover, our previous work suggests that associations between diabetes and cardiovascular disease (CVD) vary with ethnicity, with stronger associations in South Asian than in European populations 9, 11. Whether similar ethnic differences exist for associations between prediabetes and CVD risk is unclear.

The aims of the present study were to establish whether associations between prediabetes and subclinical and clinical cardiovascular disease risk differ between UK European and South Asian ethnic groups and whether any ethnic differences in associations were related to the definition of prediabetes used.

## Subjects and methods

We used cross‐sectional data from the Southall and Brent Revisited (SABRE) study, a multi‐ethnic population‐based cohort of individuals living in north‐west London 12. The South Asian participants were first‐generation migrants; 82% were born in the Indian subcontinent and 14% in East Africa. Participants aged 40–70 years (*n* = 4056) were randomly selected from age‐ and gender‐stratified general practitioner lists and workplaces at baseline (1988–1991), and followed up (2008–2011) when aged 58‐85 years (*n* = 2671). The present analysis concerns the 682 European and 520 South Asian participants who attended the follow‐up clinic. All participants gave written informed consent. Study approval was obtained from St Mary's Hospital Research Ethics Committee (07/H0712/109).

Smoking status, alcohol consumption and medication receipt were ascertained from a health and lifestyle questionnaire 12. Physical activity comprised the total weekly energy expenditure (MJ), as previously described 13. Blood pressure was obtained three times after a 15‐min rest with an Omron CEP 7050 (Omron, Tokyo, Japan); the mean of the final two readings was used in the analysis. Glucose levels, lipid profile, HbA_1c_ and C‐reactive protein levels were measured on fasting blood samples, and anthropometry performed 13, 14. In addition, an abdominal computed tomography (CT) slice was taken to measure visceral adipose tissue area 13. Participants without known diabetes underwent an oral glucose tolerance test (OGTT).

Cardiac CT scanning was performed in all participants from the ascending aorta above the level of the coronary arteries to the inferior border of the heart. Coronary artery calcification was quantified using proprietary software on a Phillips Extended Brilliance computer workstation (Phillips Healthcare, Eindhoven, The Netherlands), and calcification was defined as an area >1 mm^2^ of density >130 Hounsfield units. The coronary artery calcification score was calculated as the sum of all lesion scores [Agatston units (AU)]. Scans were read by a single experienced observer blinded to participant ethnicity. Interobserver reproducibility, comparing scores from a senior investigator (A.W.) and intra‐observer reproducibility were assessed initially and at intervals during follow‐up, using the same 20 CT scans. The intra‐class correlation coefficient for intra‐ and inter‐observer measurements was 0.94. Subclinical coronary heart disease (CHD) was classified as a coronary artery calcification score >400 AU 15.

Cerebral magnetic resonance imaging (MRI) scans provided data on brain infarcts ≥3 mm, a subclinical measure of cerebrovascular disease. We used an MRI scanning and scoring protocol based on that of the Cardiovascular Health Study 16. Whole‐brain scans included sagittal T1‐weighted images and axial T1‐weighted, proton density and T2‐weighted images of 5‐mm thickness, with no gaps. Thin‐section 3‐mm axial fluid attenuated inversion recovery (FLAIR) and coronal 1.5‐mm three‐dimensional T1‐weighted gradient echo images were also obtained. Scans were performed using a General Electric 1.5T or 3T scanners. Only infarcts of ≥3 mm were assessed, as smaller lesions are less reproducible 14. Inter‐ and intra‐observer reproducibility were evaluated for the presence of brain infarcts ≥3 mm on 44 scans; inter‐observer κ was 0.68 and intra‐observer κ was 0.79.

We used three classification systems to define glycaemic status for participants without existing diabetes. Firstly, WHO 1999 criteria were used to define prediabetes [either impaired fasting glycaemia (fasting glucose ≥6.1 mmol/l and <7.0 mmol/l) or impaired glucose tolerance (2‐h OGTT plasma glucose ≥7.8 mmol/l and <11.1 mmol/l)] and new diabetes (fasting glucose ≥7.0 mmol/l or 2‐h OGTT plasma glucose ≥11.1 mmol/l] 3. Secondly, glycaemic categories according to the International Expert Committee (IEC) 2009 criteria 2 were based on the following HbA_1c_ thresholds: prediabetes, HbA_1c_ ≥42 mmol/mol (6.0%) but < 48 mmol/mol (6.5%); new diabetes, HbA_1c_ ≥48 mmol/mol (6.5%). We also studied glycaemia according to the American Diabetes Association (ADA) 2014 recommendations 4, which advocate HbA_1c_ thresholds of ≥39 mmol/mol (5.7%) but <48 mmol/mol (6.5%) for prediabetes and ≥48 mmol/mol (6.5%) for new diabetes. Analyses were conducted using the IEC HbA_1c_ prediabetes threshold unless otherwise stated. Pre‐existing diabetes was identified from primary care record review or participant questionnaire (recall of physician‐diagnosed diabetes plus diagnosis year or diabetes medication).

Coronary heart disease was defined firstly from primary care record review adjudicated by two clinicians, as per Anglo‐Scandinavian Cardiac Outcomes Trial (ASCOT) criteria 17. Additionally, International Classification of Disease (ICD)‐9 codes 410‐415 and ICD‐10 codes I200‐I259 from Hospital Episode Statistics and codes K401‐K469, K491‐K504, K751‐K759 and U541 from the Office of Populations and Surveys classification of interventions and procedures identified CHD. For stroke, primary care data were reviewed in a similar manner to that used for CHD, with diagnoses made again according to ASCOT criteria 17. Stroke was also ascertained from participant report of physician‐diagnosed stroke with duration of symptoms ≥ 24 h and from Hospital Episode Statistics (diagnostic ICD‐9 codes 430 to 439 or ICD‐10 codes I600 to I698).

We examined descriptive statistics for demographics, cardiometabolic risk factors, medication use and CVD outcomes by ethnicity and glycaemic status. Logistic and linear regression techniques determined age‐ and sex‐adjusted group differences. Glycaemic status by OGTT or HbA_1c_ criteria was contrasted graphically in each ethnic group, using OGTT then HbA_1c_ categories in turn as the referent groups. To enhance power and address potential ethnic presentation biases, we combined established and subclinical disease outcomes for CHD and cerebrovascular disease (subclinical disease defined as coronary artery calcification >400 AU and brain infarct ≥3 mm, respectively). In addition, we created a composite CVD prevalence outcome (comprising CHD or cerebrovascular disease). Each outcome was examined by glycaemic status and ethnicity.

Following this, associations between glycaemic status and CHD, cerebrovascular disease or CVD were evaluated using age‐ and sex‐adjusted logistic regression models within each ethnic group. We then further adjusted these models for potential confounders (smoking, systolic blood pressure, triglycerides and waist–hip ratio), selected on the basis of significant associations with any CVD indicator and either prediabetes measure. We explored replacing waist–hip ratio with the more direct measure of visceral adipose tissue on CT in multivariate models, but this made little qualitative difference to our findings, so we present data adjusted for waist–hip ratio as the more familiar measure. To gauge the relative merit of each prediabetes measure for the prediction of CVD in each ethnic group, we compared C‐statistics for age‐ and sex‐adjusted models of CVD with models with the addition of either prediabetes measure.

Analyses were repeated using ADA HbA_1c_ criteria for prediabetes. We further adjusted multivariable models for medication use (as a sensitivity analysis because of data sparsity). Analyses were conducted in stata 13 (College Station, TX, USA), with *P* values <0.05 taken to indicate statistical significance.

## Results

Overt diabetes was more prevalent, and waist–hip ratio and systolic blood pressure higher, while HDL cholesterol was lower in the South Asian group than in the European group (Table 1). Irrespective of ethnic group, increasing glycaemia was associated with worsening of cardiometabolic risk factors (Table S1).

**Table 1:** Characteristics of participants in the Southall and Brent Revisited study, by ethnicity

	European ethnicity	South Asian ethnicity	*P* b
Number of participants	682	520	–
Median (IQR) age, years	70 (65–75)	68 (64–73)	0.006
Female sex, *n* (%)	153 (22)	78 (15)	0.001
Ever smoked	426 (63)	113 (22)	<0.001
Median (IQR) alcohol consumption, units/ week	5 (1–14)	4 (1–8)	<0.001
Median (IQR) physical activity, MJ/ week	9.4 (6.8–12.1)	8.8 (5.8–11.7)	0.001
Mean ± sd waist–hip ratio	0.97 ± 0.07	1.00 ± 0.07	<0.001
Mean ± sd BMI, kg/m^2^	28 ± 5	26 ± 4	<0.001
Mean ± sd systolic blood pressure, mmHg	138 ± 17	142 ± 18	<0.001
Mean ± sd diastolic blood pressure, mmHg	77 ± 10	76 ± 10	0.009
Mean ± sd HDL cholesterol, mmol/l	1.4 ± 0.4	1.3 ± 0.3	0.001
Median (IQR) triglycerides, mmol/l	1.1 (0.9–1.6)	1.2 (0.9–1.7)	0.09
Median (IQR) C‐reactive protein, mmol/l	1.7 (0.8–3.6)	1.4 (0.7–3.0)	0.01
Anti‐hypertensive medication, *n* (%)	371 (54)	395 (76)	<0.001
Lipid‐lowering medication, *n* (%)	330 (48)	354 (68)	<0.001
Glycaemic status: OGTT, *n* (%)			
Normoglycaemia	377 (55)	201 (39)	<0.001
Prediabetes	172 (25)	99 (19)	0.01
Diabetes^a^	133 (20)	220 (42)	<0.001
Glycaemic status: HbA1c, *n* (%)			
Normoglycaemia	374 (55)	136 (26)	<0.001
Prediabetes	177 (26)	146 (28)	0.37
Diabetes^a^	131 (19)	238 (46)	<0.001
CHD, *n* (%)			
Clinical CHD	142 (21)	189 (36)	<0.001
Coronary artery calcification >400 AU	119 (23)	72 (22)	0.79
Clinical CHD or coronary artery calcification^c^ >400 AU	261 (38)	258 (50)	<0.001
Cerebrovascular disease, *n* (%)			
Stroke	32 (5)	25 (5)	0.68
Brain infarct ≥3 mm	126 (21)	94 (19)	0.92
Stroke or brain infarct ≥3 mm	141 (23)	105 (21)	0.94
CHD or cerebrovascular disease, *n* (%)	335 (49)	296 (57)	0.001

AU, Agatston units; CHD, coronary heart disease; IFG, impaired fasting glucose; IGT, impaired glucose tolerance; IQR, interquartile range; OGTT, oral glucose tolerance test.

a Includes pre‐existing and newly diagnosed diabetes. Prediabetes by OGTT criteria comprised IFG and/or IGT.

b Age and sex‐adjusted *P* value for ethnic difference, when compared with European participants.

c Measured in participants without known CHD.

In the South Asian but not the European participants, there was a 1.5‐fold increase in the prevalence of prediabetes when HbA_1c_ rather than OGTT criteria were used (Table 1). When ADA HbA_1c_ thresholds were used rather than OGTT criteria, the prevalence of prediabetes doubled for both European and South Asian participants (Table S2). South Asian participants had more clinical CHD and overall CVD (CHD + cerebrovascular disease) than European participants, although there were no differences between the ethnic groups in the prevalence of subclinical CHD or subclinical and clinical cerebrovascular disease (Table 1).

**Figure 1:**
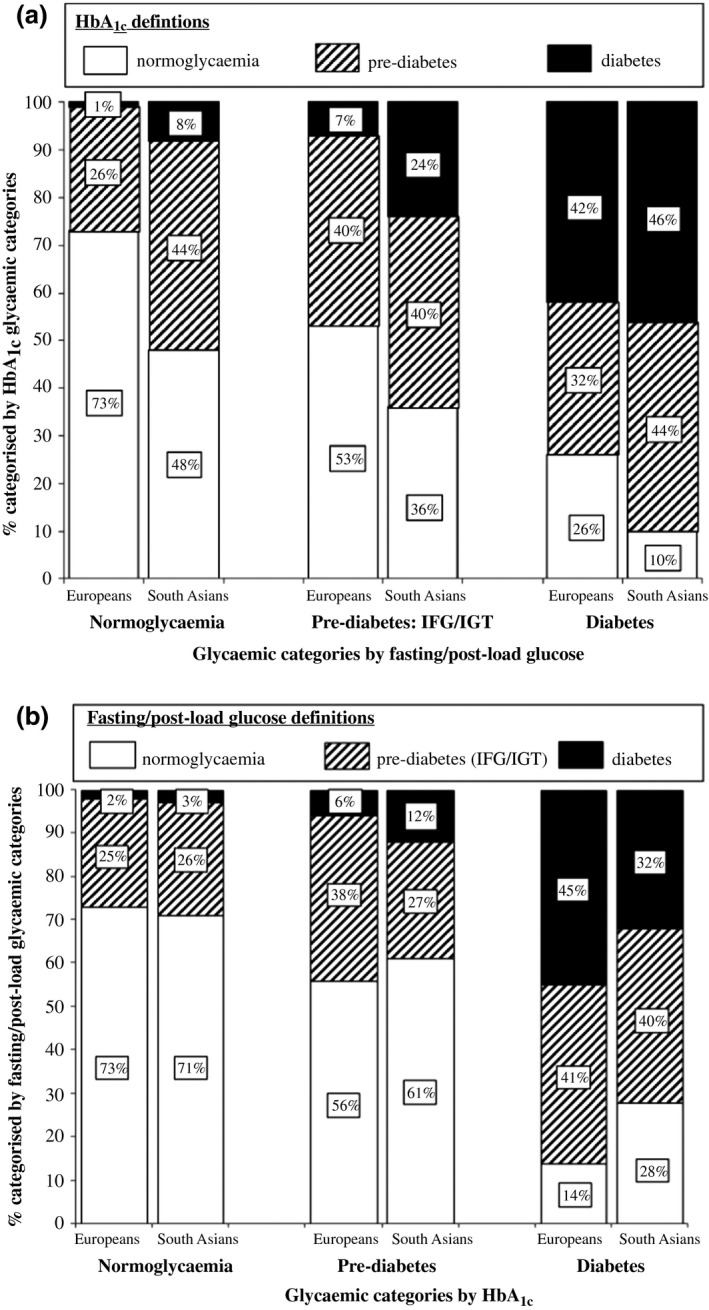
(a) Glycaemia categorized by oral glucose tolerance test (OGTT) vs. HbA_1c_, by ethnicity. Diabetes comprises newly diagnosed cases only. (b) Glycaemia categorized by HbA_1c_ vs. OGTT, by ethnicity. Diabetes comprised newly diagnosed cases only.

Differences in classification were examined by comparing glycaemic status according to either diagnostic criterion, using OGTT categories as the index classification, and excluding those with existing diabetes (Fig. 1a). With the exception of normoglycaemia in Europeans, agreement was poor (40–49%) for all categories in all groups, with a tendency for South Asian participants to be placed in a more adverse glycaemic category by HbA_1c_ classification. When HbA_1c_ categories were used as the reference (Fig. 1b), there was good agreement (71–75%) for normoglycaemia in both ethnic groups, but poor agreement for prediabetes and diabetes, especially for South Asian participants.

In the European group, CHD, cerebrovascular disease and CVD were more prevalent in prediabetes (by either criterion) than participants with normoglycaemia (Table 2). A similar, but less pronounced, pattern was seen in South Asian participants. This was even less marked when we examined disease prevalence by ADA HbA_1c_ thresholds, in both the European and South Asian groups (Table S3). When subclinical and clinical CVD measures were examined separately, similar patterns were seen (Table S4). Notably, the prevalence of clinical CHD in the South Asian participants with normoglycaemia was similar to that observed in the European participants with prediabetes.

**Table 2:** Cardiovascular disease prevalence by ethnicity and glycaemic status in the Southall and Brent Revisited study

Atherosclerosis measure	Glycaemia measure	European group, *n* (%)			South Asian group, *n* (%)		
		Normoglycaemia	Prediabetes	Diabetes	Normoglycaemia	Prediabetes	Diabetes
CHD: clinical or coronary artery calcification score >400 AU	OGTT	116 (31)	71 (41)*	74 (56)***	76 (38)	47 (47)	135 (61)***
	HbA_1c_	104 (28)	78 (44)**	79 (60)***	49 (36)	60 (41)	149 (63)***
Cerebrovascular disease: stroke or brain infarct ≥3 mm	OGTT	70 (21)	41 (26)*	30 (27)	29 (15)	15 (16)	61 (31)**
	HbA_1c_	63 (18)	47 (31)*	31 (27)	18 (14)	28 (20)	59 (27)*
CHD or cerebrovascular disease	OGTT	155 (41)	94 (55)**	86 (65)***	90 (45)	51 (52)	155 (70)***
	HbA_1c_	140 (37)	106 (60)***	89 (68)***	60 (44)	71 (49)	165 (69)***

AU, Agatston units; CHD, coronary heart disease; IFG, impaired fasting glucose; IGT, impaired glucose tolerance; OGTT, oral glucose tolerance test.

Sex‐ and age‐adjusted *P* value for difference between prediabetes or diabetes and normoglycaemia: ***p < 0.001, **p < 0.01, *p < 0.05.

Diabetes includes pre‐existing and newly diagnosed diabetes by the relevant criterion. Prediabetes by OGTT criteria comprised IFG and/or IGT.

In age‐ and sex‐adjusted models of CHD or cerebrovascular disease, prediabetes by either criterion appeared to be related to disease risk in the European group **(**Table 3). Accounting for smoking, systolic blood pressure, triglycerides and waist–hip ratio attenuated these associations, but risks remained elevated for people with prediabetes and statistical significance was retained for HbA_1c_‐defined prediabetes. Prediabetes by either criterion was associated with overall CVD, with associations being of higher magnitude when defined by HbA_1c_ rather than OGTT. By contrast, in South Asian participants, non‐significant associations were present between OGTT‐defined prediabetes only and CHD [odds ratio 1.39 (95% CI 0.84, 2.29)] and HbA_1c_‐defined prediabetes only and cerebrovascular disease [odds ratio 1.41 (95% CI 0.72, 2.76)]). Prediabetes by either criterion appeared unrelated to CVD in this group. Associations between prediabetes by HbA_1c_ and CVD were greater in the European than in the South Asian group (ethnicity interaction *P* = 0.04, respectively). Analyses using ADA HbA_1c_ thresholds showed similar patterns to the main results (Table S5).

**Table 3:** Multivariable models of cardiovascular disease by ethnicity and glycaemic status in the Southall and Brent Revisited study

Glycaemia measure		European group			South Asian group		
	Model	Normoglycaemia	Prediabetes	Diabetes	Normoglycaemia	Prediabetes	Diabetes
CHD (clinical or coronary artery calcification score >400 AU)							
OGTT	1	1	1.55 (1.05,2.29)*	2.86 (1.87,4.38)***	1	1.39 (0.84,2.29)	2.53 (1.69,3.79)***
	2	1	1.46 (0.98,2.19)	2.53 (1.61,3.98)***	1	1.41 (0.84,2.36)	2.26 (1.48,3.45)***
HbA_1c_	1	1	1.70 (1.15,2.51)**	3.79 (2.45,5.86)***	1	1.15 (0.70,1.90)	2.88 (1.84,4.53)***
	2	1	1.60 (1.07,2.39)*	3.42 (2.15,5.42)***	1	1.06 (0.64,1.77)	2.50 (1.56,4.02)***
Cerebrovascular disease (stroke or brain infarct≥3 mm)							
OGTT	1	1	1.42 (0.90,2.25)	1.28 (0.77,2.13)	1	0.91 (0.45,1.86)	2.49 (1.48, 4.18)**
	2	1	1.25 (0.63,1.88)	1.09 (0.63,1.95)	1	1.02 (0.49,4.44)	2.58 (1.49, 4.44)**
HbA_1c_	1	1	1.73 (1.10, 2.72)*	1.49 (0.90, 2.49)	1	1.41 (0.72,2.76)	2.18 (1.19, 4.00)*
	2	1	1.57 (1.00, 2.51)*	1.29 (0.75,2.22)	1	1.39 (0.69,2.78)	2.22 (1.17, 4.22)*
CHD or cerebrovascular disease							
OGTT	1	1	1.72 (1.17, 2.54)**	2.60 (1.68, 4.00)***	1	1.19 (0.72,1.98)	2.89 (1.91, 4.39)***
	2	1	1.58 (1.07, 2.36)*	2.35 (1.47, 3.73)***	1	1.20 (0.71, 2.02)	2.64 (1.71, 4.08)***
HbA_1c_	1	1	2.10 (1.43, 3.08)***	3.39 (2.18, 5.28)***	1	1.10 (0.67,1.79)†	2.79 (1.77, 4.39)***
	2	1	1.95 (1.31, 2.91)**	3.15 (1.96, 5.05)***	1	1.00 (0.61,1.66)†	2.63 (1.64, 4.22)***

AU, Agatston units; CHD, coronary heart disease; IFG, impaired fasting glucose; IGT, impaired glucose tolerance; OGTT, oral glucose tolerance test.

Data are odds ratios (95% CI) for the presence of cardiovascular disease.

***P < 0.001, **P < 0.01, *P < 0.05 for difference between prediabetes or diabetes and normoglycaemia.

†*P* < 0.05 for ethnic difference, when compared with Europeans. Diabetes includes pre‐existing and newly diagnosed diabetes by the relevant criterion. Prediabetes by OGTT criteria comprised IFG and/or IGT. Model 1: adjusted for age + sex; model 2: adjusted for age + sex + smoking status + systolic blood pressure + triglycerides (log‐transformed)) + waist–hip ratio.

When either measure of prediabetes was added to age‐ and sex‐adjusted models of CVD, there were no improvements in the C‐statistic for South Asian participants; however, addition of either OGTT‐ or HbA_1c_‐defined prediabetes to models marginally improved the C‐statistic in Europeans, more so for the latter [from 0.695 (95% CI 0.632, 0.758) to 0.705 (95% CI 0.642, 0.767)] with addition of OGTT‐defined impaired glucose regulation (*P* = 0.20) or 0.708 (95% CI 0.646, 0.769] with addition of HbA_1c_‐defined impaired glucose regulation (*P* = 0.008).

Finally, further adjustment of the multivariable models for receipt of antihypertensive or lipid‐lowering medication (Table S6) did not alter the main results.

## Discussion

There was a marked ethnic variation in prediabetes by different diagnostic criteria 2, 3, 4, with a higher prevalence in South Asian but not European participants, when prediabetes was defined by HbA_1c_ rather than OGTT criteria. Furthermore, prediabetes appeared to relate differently to cardiovascular disease according to diagnostic criteria and ethnicity; HbA_1c_‐defined prediabetes was associated with CHD and cerebrovascular disease in the European group, but with only cerebrovascular disease (non‐significantly) in the South Asian group. Furthermore, there was some evidence of a weak association between OGTT‐defined prediabetes and CHD in the European and South Asian groups. Prediabetes by either criterion was associated with total CVD (CHD + cerebrovascular disease) in the European but not the South Asian group.

When HbA_1c_, as opposed to OGTT, criteria were used to define prediabetes, South Asian participants had a higher prevalence of prediabetes, reflecting previous work 5. This may indicate ethnic differences in the propensity to glycate haemoglobin 18 or in pathways to overt hyperglycaemia 19.

Reflecting established ethnic differences in CVD, the South Asian group had greater clinical CHD than did the European group 11; however, proportions with subclinical CHD were similar in European and South Asian participants, which might be explained by our previous finding that coronary artery calcification scores are less related to coronary artery stenosis on angiography in South Asian than in European people 20. Also of note, South Asian participants in the normoglycaemic category (by any criteria) had clinical CHD rates akin to those of the European participants in the prediabetes category, implying that even normoglycaemia is a relatively high risk state in South Asian people, and thus risk factor management at the prediabetes stage may be too late.

In the European participants, associations between prediabetes by either criteria and CHD support the argument for preventative interventions at this stage of glycaemia and are consistent with previous studies reporting the effects of IFG/IGT 7, 21, 22 and HbA_1c_ 23. This was less clear for the South Asian participants, for whom only IFG/IGT‐defined prediabetes appeared detrimental. We are unaware of any studies comparing associations between prediabetes and CHD risk in European and South Asian participants, although a study of South Asian and European participants living in Canada found similar associations between HbA_1c_ and carotid intima media thickness 24. The underlying explanations for ethnic differences in the effects of glycaemia are not clear, and probably involve a combination of genetic, epigenetic and lifestyle factors.

Associations between HbA_1c_‐defined prediabetes and cerebrovascular disease appeared similar in the two ethnic groups, although OGTT‐defined prediabetes was associated (albeit weakly) with cerebrovascular disease in the European participants only; again, explanations are not obvious. A meta‐analysis of studies examining associations between IFG or IGT and stroke suggested that IGT was modestly associated with stroke 25, but a study comparing associations between IFG or HbA_1c_‐defined prediabetes in white and black populations in the USA found that HbA_1c_‐defined prediabetes, but not IFG, was adversely related to stroke risk 26. This latter study reflects our findings to some extent, but we used more stringent HbA_1c_ thresholds (IEC, as opposed to ADA criteria), more representative of UK clinical practice 27. As far as we know, there are no studies comparing the effect of prediabetes on cerebrovascular disease in European and South Asian cohorts.

Importantly, OGTT‐, and in particular, HbA_1c_‐defined prediabetes appeared to be related to overall CVD risk in the European but not in the South Asian group. This suggests that prediabetes may be a less useful clinical indicator of overall CVD risk in this group, congruent with both the findings of our model discrimination analyses and concerns other authors have expressed 6. Explanations for this difference for HbA_1c_‐based prediabetes may lie in the greater proportions identified by this measure in these ethnic groups; that is, there is less of a differential, in terms of cardiometabolic profile, between individuals with normoglycaemia and those with prediabetes, although obviously this does not apply to OGTT‐identified individuals with prediabetes. Alternatively, the loss of differential in risk may come in the normoglycaemic group, for South Asian participants at least, in whom we showed a similar prevalence of CHD to that in European participants in the prediabetic state. Our observation that associations between prediabetes and CVD were weaker in the South Asian group than in the European group, whilst associations between diabetes itself and CVD were stronger in the South Asian group than in the European group, may suggest that South Asian people destined to transition to diabetes move faster through the prediabetes state than do European people. This speculation has support from a recent publication, suggesting that the age‐related trajectory in fasting glucose is greater in South Asian people without diabetes than in European people 28. Regardless, these findings, if substantiated by future studies, have crucial policy implications for prediabetes screening in ethnic minority groups.

Using the ADA rather than the IEC criteria to define prediabetes showed similar trends and ethnic differences in the association with CHD, stroke and CVD, although odds ratios were often weaker, probably reflecting the lower HbA_1c_ threshold for ADA‐defined prediabetes.

The strengths of the present study include the high prevalence of CVD outcomes. Additionally, by capturing subclinical and clinical disease, we avoided presentation bias, which may operate when comparing different ethnic groups. There were no ethnic differences in survivorship (*P* = 0.32) or follow‐up participation (*P* = 0.62) of the cohort, suggesting survivor bias is unlikely to have greatly affected the ethnic differences we found. Furthermore, numbers of events were similar in each group, therefore, differences in power by ethnicity are unlikely to explain the lack of key associations in the South Asian participants. High rates of medication usage in this elderly population may have altered associations, although adjustment for medication use did not greatly alter results and the inclusion of participants in receipt of medication is likely to render the findings more generalizable to the elderly population as a whole. Measurement error may have been a source of bias, particularly regarding glucose and HbA_1c_ measurement; there is not insubstantial biological variation in both measures 4. The present study only examined cross‐sectional associations; therefore, we cannot infer causality and further longitudinal research is required to replicate the findings.

In summary, the choice of diagnostic criterion for prediabetes markedly influences both the number of individuals identified in the South Asian group and the CVD risk conferred by prediabetes. When considering overall CVD risk, prediabetes was associated with risk in the European but not in the South Asian group; therefore, the use of prediabetes as a marker of CVD risk may be less clinically meaningful in this group. Of importance when considering CHD risk estimation is the observation that South Asian individuals with normoglycaemia have similar risks of CHD to European individuals with prediabetes. These novel findings have implications for CVD risk stratification and targeting of interventions in different ethnic groups, and further work is needed to substantiate them.

## Funding sources

The study was funded at baseline by the UK Medical Research Council, Diabetes UK and the British Heart Foundation and at follow‐up by the Wellcome Trust and British Heart Foundation. The authors also wish to acknowledge the NIHR Imperial Biomedical Research Centre for structural support regarding the data collection at Imperial Trust NHS Campus and MRC Epidemiology Unit core support (NGF) MC_UU_12015/5. Funders played no role in the study design, conduct or analysis, or in the decision to submit the manuscript for publication. The SABRE study group is entirely independent from the funding bodies.

## Competing interests

None declared.

## Supporting information


**Table S1** Characteristics of participants in the Southall and Brent Revisited study, by ethnicity and glycaemic status.
**Table S2** Glycaemic status of participants in the Southall and Brent Revisited study using American Diabetes Association criteria for prediabetes [HbA_1c_ ≥39 mmol/mol (5.7%) to <48 mmol/mol (6.5%)], by ethnicity.
**Table S3** Cardiovascular disease by ethnicity and glycaemic status in the Southall and Brent Revisited study, using American Diabetes Association criteria for prediabetes [≥39 mmol/mol (5.7%) and <48 mmol/mol (6.5%)], by ethnicity.
**Table S4** Clinical or subclinical cardiovascular disease by ethnicity and glycaemic status in the Southall and Brent Revisited study.
**Table S5** Multivariable models of cardiovascular disease by ethnicity and glycaemic status; ADA prediabetes thresholds.
**Table S6** Multivariable models of cardiovascular disease by ethnicity and glycaemic status in the Southall and Brent Revisited study, with further adjustment for medication use.
