# Supplementary material for: Ethnic differences in cross‐sectional associations between impaired glucose regulation, identified by oral glucose tolerance test or HbA1c values, and cardiovascular disease in a cohort of European and South Asian origin
Source: Diabet Med. 2015 Oct 19;33(3):340–7. doi: 10.1111/dme.12895 (PMC4740925; doi:10.1111/dme.12895)
Supplement: Supplementary file 1 — Table S1 Characteristics of participants in the Southall and Brent Revisited study, by ethnicity and glycaemic status. Table S2 Glycaemic status of participants in the Southall and Brent Revisited study using American Diabetes Association criteria for prediabetes [HbA1c ≥39 mmol/mol (5.7%) to <48 mmol/mol (6.5%)], by ethnicity. Table S3 Cardiovascular disease by ethnicity and glycaemic status in the Southall and Brent Revisited study, using American Diabetes Association criteria for prediabetes [≥39 mmol/mol (5.7%) and <48 mmol/mol (6.5%)], by ethnicity. Table S4 Clinical or subclinical cardiovascular disease by ethnicity and glycaemic status in the Southall and Brent Revisited study. Table S5 Multivariable models of cardiovascular disease by ethnicity and glycaemic status; ADA prediabetes thresholds. Table S6 Multivariable models of cardiovascular disease by ethnicity and glycaemic status in the Southall and Brent Revisited study, with further adjustment for medication use. [file DME-33-340-s001.docx]

**Table S1. Characteristics of participants in the SABRE study, by ethnicity and glycaemic status.**

|  | ***Glycaemic category; OGTT*** | | | ***Glycaemic category; HbA_1c_ (IEC criteria)*** | | |
| --- | --- | --- | --- | --- | --- | --- |
|  | **NG** | **Pre-diabetes** | **Diabetes** | **NG** | **Pre-diabetes** | **Diabetes** |
| **Europeans** | | | | | | |
| n | 377 | 172 | 133 | 374 | 177 | 131 |
| Age, years | 69 (64-74) | 70 (65-75) | 70 (66-75)* | 68(64-74) | 71 (66-76)* | 70 (65-75)* |
| Female sex | 86 (23) | 35 (20) | 32 (24) | 95 (25) | 32 (18) | 26 (20) |
| Ever smoked | 226 (60) | 113 (66) | 87 (66) | 224 (60) | 115(65) | 87 (67) |
| Alcohol, units/wk | 6(2-14) | 5(2-14) | 3(0-10)*† | 6(2-14) | 4(1-12) | 4(0-12)* |
| Physical activity, MJ/ week | 9.5(6.8-12.5) | 9.7(7.6-12.4) | 7.8(5.8-10.8)*† | 9.8(6.9-12.7) | 8.8(6.8-11.7) | 7.8(5.8-10.7)*† |
| BMI, kg/m^2^ | 27±4 | 28±4* | 31±6*† | 26±4 | 26±4 | 27±4* |
| Waist/ hip ratio | 0.96±0.07 | 0.97±0.07 | 1.00±0.08*† | 0.95±0.07 | 0.98±0.07* | 1.00±0.07*† |
| SBP, mmHg | 136±17 | 142±18* | 139±18* | 137±17 | 140±19 | 138±18 |
| DBP, mmHg | 77±9 | 79±11* | 77±10 | 77±10 | 77±10 | 77±10 |
| HDL, mmol/l | 1.4±0.4 | 1.3±0.3* | 1.2±0.3*† | 1.4±0.4 | 1.4±0.3 | 1.2±0.3*† |
| Trigs, mmol/l | 1.08 (0.82-1.43) | 1.26 (0.9-1.70)* | 1.41(0.95-2.00)* | 1.09(0.84-1.45) | 1.20(0.86-1.57) | 1.45(1.00-2.00)*† |
| CRP, mmol/l | 1.6(0.8-3.2) | 1.6(0.8-3.5) | 2.1(1.0-4.7)* | 1.5(0.7-3.1) | 1.7(0.9-3.7) | 2.2(0.9-4.7)* |
| Anti-hypertensives | 165(44) | 94(55)* | 112(84)*† | 156 (42) | 100(57)* | 115(88)*† |
| Lipid-lowering medication | 134 (36) | 92 (53)* | 104 (78)*† | 128 (34) | 93(53)* | 109 (83)*† |
| **South Asians** | | | | | | |
| n | 201 | 99 | 220 | 136 | 146 | 238 |
| Age, years | 67(64-72) | 69(65-75) | 69(64-73) | 68(63-72) | 69(65-74) | 69(64-73) |
| Female sex | 32(16) | 19(19) | 27(12) | 25(18) | 22(15) | 31(13) |
| Ever smoked | 37(19) | 15(15) | 61(28)*† | 18(13) | 35(24)* | 60(26)* |
| Alcohol, units/wk | 3(1-8) | 3(0-8) | 4(1-10) | 4(1-10) | 3(1-7) | 4(1-10) |
| Physical activity, MJ/ week | 9.5(6.-12.7) | 9.2(5.8-11.7) | 7.8(5.6-10.7)*† | 9.4(6.6-11.7) | 9.6(7.4-12.7) | 7.8(5.4-10.7)*† |
| BMI, kg/m^2^ | 27±4 | 28±4* | 32±6*† | 25±4 | 26±4 | 27±4*† |
| Waist/ hip ratio | 0.99±0.06 | 1.00±0.07 | 1.02±0.07*† | 0.98±0.07 | 1.00±0.05* | 1.02±0.08*† |
| SBP, mmHg | 140±17 | 142±17 | 144±19* | 140±19 | 141±17 | 144±19 |
| DBP, mmHg | 77±10 | 76±10 | 76±10 | 77±10 | 77±9 | 76±10 |
| HDL, mmol/l | 1.4±0.3 | 1.3±0.3* | 1.2±0.3* | 1.4±0.3 | 1.3±0.3 | 1.2±0.3*† |
| Trigs, mmol/l | 1.16(0.86-1.55) | 1.22(0.94-1.78) | 1.23(0.93-1.72)* | 1.09(0.83-1.45) | 1.19(0.88-1.61) | 1.26(0.97-1.76)* |
| CRP, mmol/l | 1.3(0.6-2.9) | 1.5(0.8-3.7) | 1.3(0.7-2.9) | 1.3(0.5-2.6) | 1.7(0.8-3.1) | 1.4(0.7-2.9) |
| Anti-hypertensives | 129(64) | 73(75) | 193(88)*† | 86(63) | 104(71) | 205(86)*† |
| Lipid-lowering medication | 117(58) | 60(61) | 177(80)*† | 71(52) | 92(63) | 191(80)*† |

Data are n (%), median (IQR) or mean ± SD. *p<0.05 pre-diabetes or diabetes vs. normoglycaemia (NG), †p<0.05 diabetes vs. pre-diabetes.

**Table S2. Glycaemic status of participants in the SABRE study using American Diabetes Association criteria for pre-diabetes (HbA_1c_ ≥39mmol/mol (5.7%) to <48mmol/mol (6.5%)), by ethnicity.**

|  | **Europeans** | **South Asians** | **p*** |
| --- | --- | --- | --- |
| Normoglycaemia | 168 (25) | 45 (9) | <0.001 |
| Pre-diabetes | 383 (56) | 237 (46) | <0.001 |
| Diabetes† | 131 (19) | 238 (46) | <0.001 |

Data are n (%). *Age and sex-adjusted p for ethnic difference. †Includes pre-existing and newly-diagnosed diabetes.

**Table S3. Cardiovascular disease by ethnicity and glycaemic status in the SABRE study, using American Diabetes Association criteria for pre-diabetes (≥39 mmol/mol (5.7%) and <48 mmol/mol (6.5%), by ethnicity.**

|  | **Europeans** | | | **South Asians** | | |
| --- | --- | --- | --- | --- | --- | --- |
| **Atherosclerosis measure** | **NG** | **Pre-diabetes** | **Diabetes** | **NG** | **Pre-diabetes** | **Diabetes** |
| Coronary heart disease  (clinical or CAC>400 Agatston units) | 43(26) | 139(36) | 79(60)*** | 17(38) | 92(39) | 149(63)** |
| Cerebrovascular disease  (stroke or brain infarct≥3mm) | 26(17) | 84(25) | 31(27) | 5(12) | 41(18) | 59(27)* |
| Coronary heart disease or cerebrovascular disease | 60(36) | 186(49)* | 89(68)*** | 20(44) | 111(47) | 165(69)** |

Data are n (%). Sex- and age-adjusted p for difference between pre-diabetes or diabetes and normoglycaemia: ***p <0.001, **p<0.01, *p<0.05. NG=normoglycaemia, diabetes includes pre-existing and newly-diagnosed diabetes. CAC=coronary artery calcification score.

**Table S4. Clinical or subclinical cardiovascular disease by ethnicity and glycaemic status in the SABRE study.**

|  |  | | **Europeans** | | | **South Asians** | | |
| --- | --- | --- | --- | --- | --- | --- | --- | --- |
| **Atherosclerosis measure** | | **Glycaemia measure** | **NG** | **Pre-diabetes** | **Diabetes** | **NG** | **Pre-diabetes** | **Diabetes** |
| CAC>400 Agatston units | | *OGTT* | 58 (18) | 28 (22) | 33 (38)*** | 20 (14) | 12 (19) | 40 (32)** |
|  |  | *HbA_1c_* | 57(18) | 27 (22) | 35 (42)*** | 14 (14) | 15 (15) | 43(33)** |
| Clinical coronary heart disease | | *OGTT* | 58 (15) | 43 (25)* | 41 (31)*** | 58 (29) | 36 (36) | 95 (43)** |
|  |  | *HbA_1c_* | 47 (13) | 51 (29)*** | 44 (34)*** | 38 (28) | 45 (31) | 106 (45)** |
| Brain infarct≥3mm | | *OGTT* | 63 (19) | 37 (24) | 26 (23) | 27 (14) | 14 (15) | 53 (27)** |
|  |  | *HbA_1c_* | 58 (17) | 40 (26) | 28 (25) | 18 (14) | 26 (18) | 50 (23) |
| Stroke | | *OGTT* | 16 (4) | 12 (7) | 4 (3) | 4 (2) | 4 (4) | 17 (8)* |
|  |  | *HbA_1c_* | 14 (4) | 14 (8) | 4 (3) | 1 (1) | 6 (4) | 18 (8)* |

Data are n (%). Sex- and age-adjusted p for difference between pre-diabetes or diabetes and normoglycaemia: ***p <0.001, **p<0.01, *p<0.05. NG=normoglycaemia, diabetes includes pre-existing and newly-diagnosed diabetes by the relevant criterion. CAC=coronary artery calcification score.

**Table S5. Multivariable models of cardiovascular disease by ethnicity and glycaemic status; ADA pre-diabetes** **cut-points.**

|  |  | **Europeans** | | | **South Asians** | | | | |
| --- | --- | --- | --- | --- | --- | --- | --- | --- | --- |
| **Coronary heart disease (clinical or CAC>400 Agatston units)** | | | | |  |  | |  | |
| **Model** | | **NG** | **Pre-diabetes** | **Diabetes** | **NG** | | **Pre-diabetes** | | **Diabetes** |
| 1 | | 1 | 1.48(0.97,2.25) | 4.18(2.50,6.97)*** | 1 | | 1.08(0.55,2.13) | | 2.86(1.45,5.63)** |
| 2 | | 1 | 1.47(0.96,2.27) | 3.81(2.21,6.54)*** | 1 | | 1.16(0.58,2.34) | | 2.74(1.35,5.57)** |
| **Cerebrovascular disease (stroke or brain infarct≥3mm)** | | | | |  |  | |  | |
| **Model** | | **NG** | **Pre-diabetes** | **Diabetes** | **NG** | | **Pre-diabetes** | | **Diabetes** |
| 1 | | 1 | 1.45(0.88,2.39) | 1.60(0.87,2.93) | 1 | | 1.80(0.48,2.00) | | 2.98(1.67,2.78)* |
| 2 | | 1 | 1.34(0.81,2.23) | 1.34(0.71,2.55) | 1 | | 1.73(0.61,4.88) | | 2.92(1.04,8.22)* |
| **Coronary heart disease or cerebrovascular disease** | | | | |  |  | |  | |
| **Model** | | **NG** | **Pre-diabetes** | **Diabetes** | **NG** | | **Pre-diabetes** | | **Diabetes** |
| 1 | | 1 | 1.52(1.02,2.25)* | 3.57(2.15,5.93)*** | 1 | | 1.15(0.59,2.26) | | 3.00(1.52,5.91)** |
| 2 | | 1 | 1.47(0.98,2.20) | 3.29(1.92,5.63)*** | 1 | | 1.24(0.62,2.49) | | 2.90(1.43,2.49)** |

Data are odds ratios (95% CI) for the presence of cardiovascular disease,***p<0.001, **p<0.01, *p<0.05 for difference between pre-diabetes or diabetes and normoglycaemia. NG=normoglycaemia. CAC=coronary artery calcification. Model 1: age + sex, model 2: age + sex + smoking + SBP + triglycerides +WHR.

**Table S6. Multivariable models of cardiovascular disease by ethnicity and glycaemic status in the SABRE study, with further adjustment for medication use.**

|  |  | **Europeans** | | | **South Asians** | | |
| --- | --- | --- | --- | --- | --- | --- | --- |
| **Coronary heart disease (clinical or CAC>400 Agatston units)** | | | | | | | |
| **Glycaemia measure** | **Model** | **NG** | **Pre-diabetes** | **Diabetes** | **NG** | **Pre-diabetes** | **Diabetes** |
| *OGTT* | 1 | 1 | 1.55(1.05,2.29)* | 2.86(1.87,4.38)*** | 1 | 1.39(0.84,2.29) | 2.53(1.69,3.79)*** |
|  | 2 | 1 | 1.18(0.76,1.82) | 1.42(0.87,2.31) | 1 | 1.37(0.79,2.37) | 1.75(1.12,2.74)* |
| *HbA_1c_* | 1 | 1 | 1.70(1.15,2.51)** | 3.79(2.45,5.86)*** | 1 | 1.15(0.70,1.90) | 2.88(1.84,4.53)*** |
|  | 2 | 1 | 1.34(0.87,2.07) | 1.80(1.09, 2.98)* | 1 | 0.96(0.56,1.64) | 1.90(1.15,3.15)* |
| **Cerebrovascular disease (stroke or brain infarct≥3mm)** | | | | | | | |
| **Glycaemia measure** | **Model** | **NG** | **Pre-diabetes** | **Diabetes** | **NG** | **Pre-diabetes** | **Diabetes** |
| *OGTT* | 1 | 1 | 1.42(0.90,2.25) | 1.28(0.77,2.13) | 1 | 0.91(0.45,1.86) | 2.49(1.48,4.18)** |
|  | 2 | 1 | 1.11(0.68,1.80) | 0.81(0.46,1.44) | 1 | 1.01(0.49,2.09) | 2.46(1.41,4.28)**† |
| *HbA_1c_* | 1 | 1 | 1.73(1.10,2.72)* | 1.49(0.90, 2.49) | 1 | 1.41(0.72,2.76) | 2.18(1.19,4.00)* |
|  | 2 | 1 | 1.44 (0.90, 2.33) | 0.95(0.53,1.68) | 1 | 1.32(0.66,2.66) | 2.02(1.05,3.89)* |
| **Coronary heart disease or cerebrovascular disease** | | | | | | | |
| **Glycaemia measure** | **Model** | **NG** | **Pre-diabetes** | **Diabetes** | **NG** | **Pre-diabetes** | **Diabetes** |
| *OGTT* | 1 | 1 | 1.72(1.17,2.54)** | 2.60(1.68,4.00)*** | 1 | 1.19(0.72,1.98) | 2.89(1.91,4.39)*** |
|  | 2 | 1 | 1.35(0.88,2.06) | 1.32(0.80,2.17) | 1 | 1.14(0.66,1.97) | 2.11(1.34,3.33)** |
| *HbA_1c_* | 1 | 1 | 2.10(1.43,3.08)*** | 3.39(2.18,5.28)*** | 1 | 1.10(1.77,4.39)† | 2.79(1.77,4.39)*** |
|  | 2 | 1 | 1.74(1.14,2.65)* | 1.67(1.00,2.80)* | 1 | 0.92(0.54,1.57) | 1.91(1.15,3.16)* |

Data are odds ratios (95% CI) for the presence of cardiovascular disease,***p<0.001, **p<0.01, *p<0.05 for difference between pre-diabetes or diabetes and normoglycaemia,†p<0.05 for ethnic difference. NG=normoglycaemia, diabetes includes pre-existing and newly-diagnosed diabetes by the relevant criterion. CAC=coronary artery calcification score. Model 1: adjusted for age + sex, model 2: adjusted for age + sex + smoking status + systolic blood pressure + triglycerides (log-transformed)) + waist/hip ratio + anti-hypertensive medication + lipid-lowering medication.
